# The impact of land cover change on the carbon stock of moist afromontane forests in the Majang Forest Biosphere Reserve

**DOI:** 10.1186/s13021-023-00243-z

**Published:** 2023-12-07

**Authors:** Semegnew Tadese, Teshome Soromessa, Abreham Berta Aneseye, Getaneh Gebeyehu, Tomasz Noszczyk, Mengistie Kindu

**Affiliations:** 1 Centre for Environmental Science, College of Natural and Computational Sciences, Addis Ababa University, Addis Ababa, Ethiopia; 2 Department of Natural Resource Management, College of Agriculture and Natural Resource, Wolkite University, Welkite, Ethiopia; 3 Department of Biology, College of Natural and Computational Sciences, Injibara University, Injibara, Ethiopia; 4 Department of Land Management and Landscape Architecture, Faculty of Environmental Engineering and Land Surveying, University of Agriculture in Krakow, 21 Mickiewicza Street, Krakow, 31-120 Poland; 5 TUM School of Life Sciences Weihenstephan, Institute of Forest Management, Technical University of Munich, Hans-Carl-von-Carlowitz-Platz 2, 85354 Freising, Germany

**Keywords:** Land use/cover, Carbon stock, Environment and disturbance factors, InVEST model, Africa

## Abstract

**Backgorund:**

Forest plays an important role in the global carbon cycle by sequestering carbon dioxide and thereby mitigating climate change. In this study, an attempt was made to investigate the effects of land use/land cover (LULC) change (1989–2017) on carbon stock and its economic values in tropical moist Afromontane forests of the Majang Forest Biosphere Reserve (MFBR), south-west Ethiopia. Systematic sampling was conducted to collect biomass and soil data from 140 plots in MFBR. The soil data were collected from grassland and farmland. InVEST modelling was employed to investigate the spatial and temporal distribution of carbon stocks. Global Voluntary Market Price (GVMP) and Tropical Economics of Ecosystems and Biodiversity (TEEB) analysis was performed to estimate economic values (EV) of carbon stock dynamics. Correlation and regression analyses were also employed to identify the relationship between environmental and anthropogenic impacts on carbon stocks.

**Results:**

The results indicated that the above-ground carbon and soil organic carbon stocks were higher than the other remaining carbon pools in MFBR. The mean carbon stock (32.59 M tonne) in 2017 was lower than in 1989 (34.76 Mt) of MFBR. Similarly, the EV of carbon stock in 2017 was lower than in 1989. Elevation, slope, and harvesting index are important environmental and disturbance factors resulting in major differences in carbon stock among study sites in MFBR.

**Conclusions:**

Therefore, the gradual reduction of carbon stocks in connection with LULC change calls for urgent attention to implement successful conservation and sustainable use of forest resources in biosphere reserves.

## Background

Forests play an important role in the global carbon cycle, sequestering carbon dioxide (CO_2_) and thereby mitigating climate change [1, 2]. They control climate change by sinking over 200 billion metric tons of carbon a year and converting atmospheric carbon into biomass through photosynthesis [3, 4]. They are significant carbon sinks, accounting for half of the above-ground biomass in vegetation [2, 5]. Moreover, the current carbon stock in global forests is estimated at 861 Gt of carbon, of which 363 and 383 Gt of carbon are stored in the living biomass and soil (up to 1 m), respectively [6–8].

The global carbon cycle has sparked the most interest in recent years as it became clear that rising levels of CO_2_ in the atmosphere cause rapid changes in global climate [9, 10]. In the international dialogue, issues such as biodiversity loss, ozone layer depletion, and desertification have taken a central stage [11]. Humans exert significant pressure on the carbon cycle through the use of large amounts of oil, gasoline, and coal, as well as deforestation and land degradation [12, 13]. Deforestation and land degradation are also the major sources of anthropogenic greenhouse gas (GHG) emissions in most tropical countries [14, 15]. Changes in land use/land cover (LULC) are reducing globally significant carbon storage that is currently sequestering CO_2_ from the atmosphere, which makes them critical to long-term climate stability [16, 17]. Every year, tropical deforestation accounts for 15–25% of global GHG emissions [15]. Liu, Van Dijk [18] indicated that between 1993 and 2012, the global Above-Ground Carbon (AGC) declined at a rate of − 0.07 PgC/yr due to the loss of tropical forest area. Pan, Birdsey [8] reported that the global soil organic carbon (SOC) decreased by 7.7% (12.7 PgC) between 1990 and 2007, owing primarily to tropical deforestation. Specifically, timber extraction and logging are accountable for over half of forest degradation (52%), followed by fuel wood extraction and charcoal production (31%), induced fire (9%), and overgrazing (7%) in the tropics [19]. This showed that forest degradation and deforestation are the main sources of GHG emissions in most tropical countries.

The InVEST models typically quantify and investigate trade-offs associated with alternative management options as well as indicate areas where natural capital projects can improve land conservation and development [20–22]. InVEST models are spatially explicit (they use maps as input and output) and produce results in either biophysical (e.g., tons of carbon sequestered) or economic terms (e.g., the net present value of that sequestered carbon) [23, 24]. Such a model effectively estimates carbon stock in the landscape ecosystem using carbon pools and LULC classes as input data [25]. Therefore, it provides carbon stock estimates over a large area for trend analysis [21, 26].

Carbon valuation is a monetary estimation of carbon related to small changes in emissions of CO_2_ [27, 28]. Carbon valuation is essential for evaluating the relative positive effects of climate mitigation and adaptation policy over time [29–32]. Future carbon benefits are strongly connected to risk management concerns because future values are affected by the chance that benefits may not emerge as expected [33, 34]. Carbon valuation is complicated, and multiple methodologies and sources are used depending on whether a societal or market perspective is used [24, 35]. Although there is no single accepted method for calculating the social value of carbon [30, 36], the Global Voluntary Market Price (GVMP) and Tropical Economics of Ecosystems and Biodiversity (TEEB) databases are used to change carbon stocks as economic terms [37, 38].

Moist Afromontane forests provide a variety of ecosystem services, such as watershed protection, groundwater regulation, food control, prevention of soil erosion, provision of non-timber forest products, and climate change mitigation [39–41]. More specifically, the Majang Forest Biosphere Reserve (MFBR) is one of the recently registered forest biosphere reserves in southern Ethiopia, which is part of the remnants of moist Afromontane forests that continue to provide essential services for people’s livelihood [42]. Anthropogenic activities have gradually degraded these moist Afromontane forests over time because they have not been managed sustainably [43–45]. Moreover, estimating changes in carbon stock and its economic value due to changes in forest cover has not been investigated yet. Understanding this encourages decision-makers to create a carbon credit negotiation and sustainable development and conservation of MFBR. Therefore, the aims of this study were “to” (i) examine the change in carbon stocks due to forest cover change over the last 30 years, (ii) map the carbon stock dynamics and its economic value, and (iii) analyse the impacts of environmental and disturbance factors on carbon stocks.

## Methods

### Study area

**Fig. 1:**
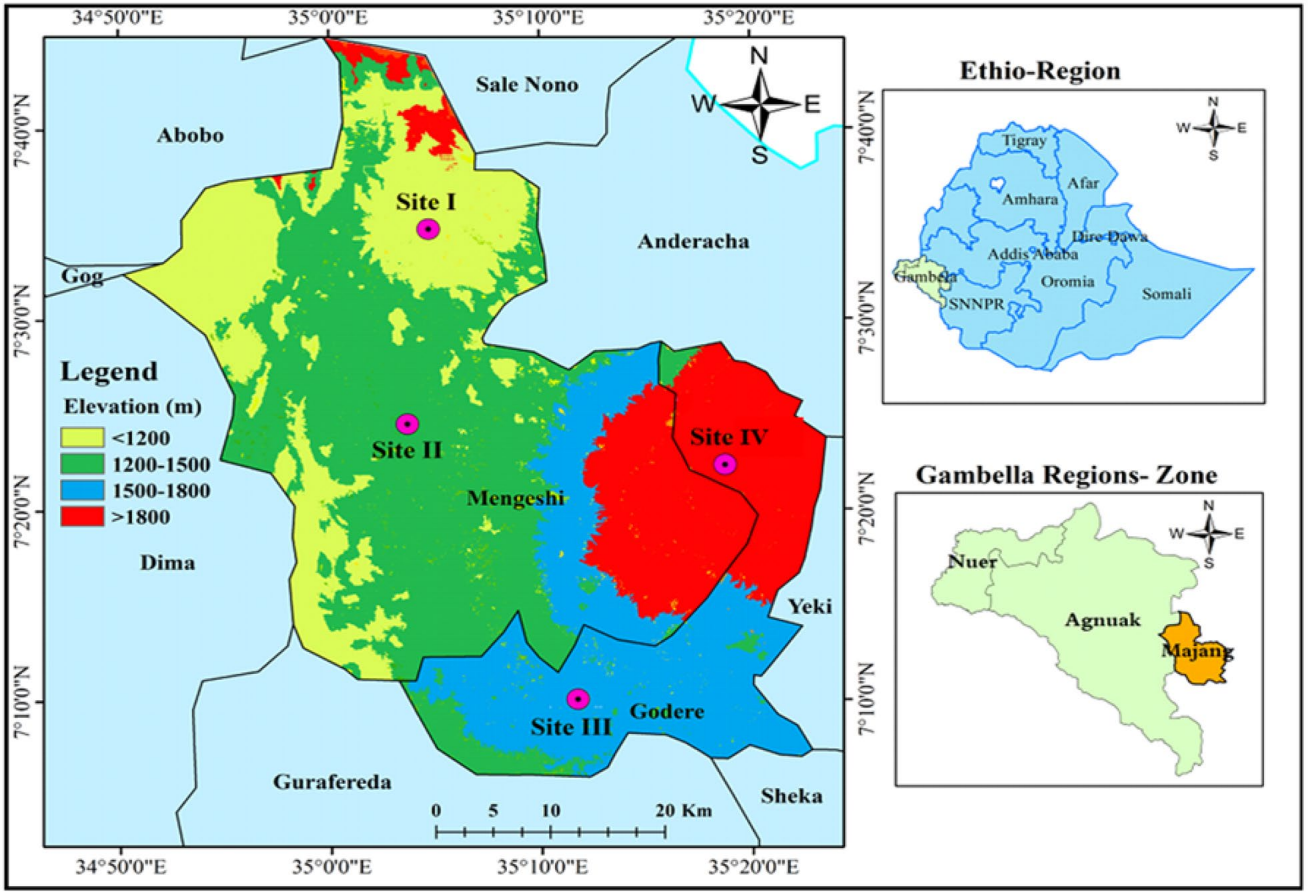
Location of the study sites (site I–IV) (https://earthexplorer.usgs.gov)

The study was conducted in the Majang Forest Biosphere Reserve (MFBR), situated in the Majang Zone, *Gambella People National Regional State* of Ethiopia. It has unique biogeography and shares a boundary with Sale Nono Woreda of the Oromia Regional State; Anderacha, Yeki, Sheka, and Gurafereda Woreda of the Southern Nations, Nationalities, and Peoples’ Region (SNNPR). It covers a total area of 233,254 ha of forest and agricultural land and rural settlements and towns (Fig. 1). MFBR is located between the latitudes of 07° 08′ 00″ N and 07° 50′ 00″ N, and the longitudes of 34° 50′ 00″ E and 35° 25′ 00″ E, with elevations ranging from 562 to 2444 m above sea level (m a.s.l.).

It is distinctive biogeography and shares a boundary with Illubabor Zone of Oromia Regional State; Sheka and Bench-Maji Zones of the Southern Nations, Nationalities, and People Region (SNNPR).

**Fig. 2:**
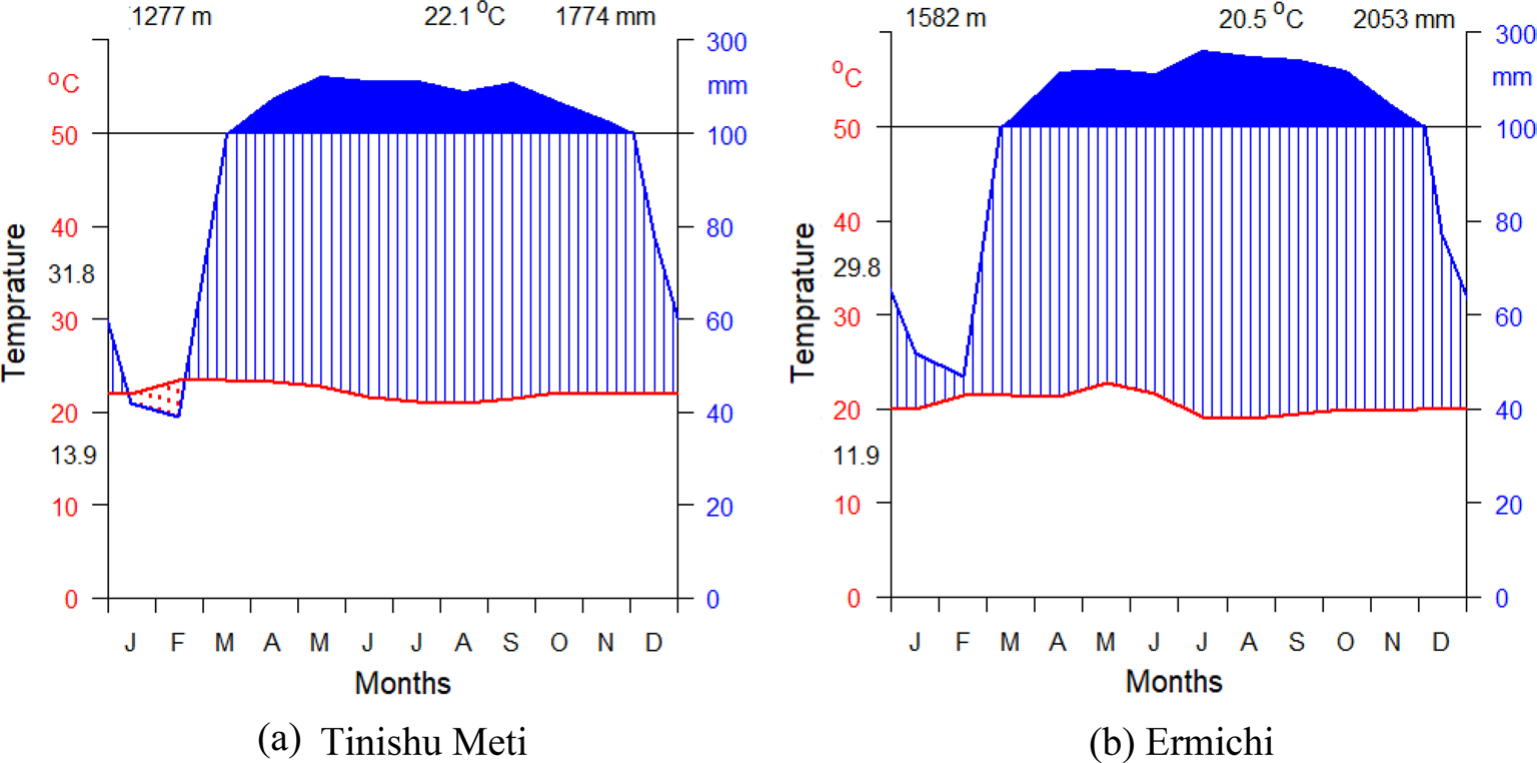
Mean monthly temperature and rainfall recorded at **a** Tinishu Meti (1987–2017) and **b** Ermichi (1987–2017) (NMSA 2018)

The climate in the area is generally hot and humid, which is marked on most rainfall maps of Ethiopia as the wettest part of the country. The annual average rainfall and temperature are 1774 mm and 22.1 °C, the mean annual minimum and maximum monthly temperature ranges between 13.9 and 31.8 °C in Tinishu Meti metrological station respectively. The annual average rainfall and temperature are 2053 mm and 20.5 °C, the mean annual minimum and maximum monthly temperature ranges between 11.8 and 29.7 °C in Ermichi Metrological station respectively (Fig. 2).

The vegetation in the area is divided into several categories based on its life forms, including high natural forests, woodlands, bush lands, and grasslands. *Euphorbiaceae, Rubiaceae*, and *Moraceae* were the most prevalent families in MFBR, with 13 species (8%), nine genera (7.8%), twelve species (7.4%) and eight genera (7%), and ten species (6.1%), and five genera (4.3%), respectively [46].

### Sampling design

A systematic sampling design was used to arrange quadrats and transects as well as to collect vegetation data [47]. The study area was stratified into four sites using Digital Elevation Model (DEM) in the Arc GIS software. These were site I (< 1200 m a.s.l.), site II (1200–1500 m a.s.l.), site III (1500–1800 m a.s.l.) and site IV (> 1800 m a.s.l.) (Table 1; Fig. 1). The number of transect lines varied among study sites. A total of 140 quadrats were established for vegetation and forest soil data collection. Farmland (40) and grassland (40) soil samples were acquired from adjoining forestland in each study site of the MFBR.

**Table 1:** Topographic and soil characteristics of the study sites

Study site	Ele (m)	Slo (°)	pH	TN (%)	P (ppm)	Area (ha)	SP
Site I	1042 ± 42	5.3 ± 0.4	6.6 ± 0.4	0.32 ± 0.1	16.2 ± 1.1	22,826.1	40
Site II	1365 ± 24	5.4 ± 0.4	6.3 ± 0.4	0.24 ± 0.8	17.48 ± 1.4	25,220.5	45
Site III	1635 ± 24	7.2 ± 0.5	6.0 ± 0.5	0.14 ± 0.1	19.02 ± 1.8	14,053	30
Site IV	2011 ± 42	11.1 ± 1.2	5.8 ± 0.4	0.11 ± 0.3	20.23 ± 2.4	11,783.5	25

*TN* total nitrogen, *P* phosphorus, *pH* soil pH, *Ele* elevation, *Slo* slope, *SP* sample plots, *ppm* part per million

The study site polygon was digitized using Google Earth by elevation classes. The quadrats’ X–Y coordinates were generated using GIS tools and loaded to a global positioning system (GPS) receiver for tracking quadrats. Later, a measuring tape was used to layout 20 × 20 m (400 m^2^) quadrats in each site in the biosphere. The sampling intervals between the transect line and the quadrats were 2 km apart. Biomass data for tree census in the tree sites were collected on 5.6 ha (4 sites = 140 quadrats). Above-ground biomass was estimated using a non-destructive sampling method by measuring the diameter at breast height (DBH), tree height, and wood density [48].

### Biomass and soil data collection

During the field data collection, the main carbon measurement activities concerned above-ground tree biomass, below-ground biomass, leaf litter, deadwood, and soil organic carbon. Individual trees with a DBH of > 5 cm [49] were measured in each plot with a calliper and measuring tape (at 1.3 m). Each tree was individually recorded, along with its species name and ID. Clinometers and a meter tape were used to measure the heights of all individual trees in the sampling quadrats. Overhanging species were excluded, but trees with trunks inside the sampling plot and branches outside were included [50].

Five rectangular subplots of 1 × 1 m were established at the four corners and centre of each main plot for litter, herbs, and soil data collection. Where the samples were large, the fresh weight of the total sample was recorded in the field, and a manageable-sized (200 g) evenly mixed subsample was brought to the laboratory to determine dry biomass and percentage carbon [51]. The biomass in the pool of leaf Litter, Grass, and Herbs (LGH) was estimated using destructive sampling. Herbaceous samples were collected by clipping and weighing all vegetation before placing it in a sample weighing bag and transporting it to the laboratory to determine the oven-dry weight of the biomass. Forest floor litter materials (dead leaves, twigs, fruit, and flowers) were collected from a 1 m^2^ area. The living components, primarily grass and herbs, were harvested and weighed as well. Dry weight was determined in laboratory samples of the materials. Within the 400 m^2^ plot, standing dead trees, fallen stems, and fallen branches with a DBH ≥ 5 cm were measured [51].

Soil samples were taken with a soil auger from the topsoil at a depth of 0–30 cm, which is recommended as the default sampling depth for soil [52]. Soil samples were taken from five different locations in each plot, four from the quadrat’s corners and one from the quadrat’s centre. A total of 220 soil samples, 140 from forestland, 40 from farmland, and 40 from grassland were collected, composited separately, labelled, and transported to the laboratory. To determine soil bulk density, the soils were collected on the centre of the quadrats using a stainless core sampler, then placed in plastic bags, and transported to the laboratory for dry weight determination. Fresh wet soil weights were measured in the field with a kitchen balance with 0.1 g precision. A composite sample of 200 g was taken from each quadrat to analyse its chemical composition [51].

### Environmental and disturbance factors

Environmental factors such as aspect, slope, and elevation were measured and recorded for each of the 140 quadrats using a Garmin GPS receiver and clinometers. Elevation was arranged into four elevation (m a.s.l.) ranges (sites I–IV), namely: 1 = 1200, 2 = 1200–1500, 3 = 1500–1800 and 4 ≥ 1800. The slope range was classified into three major slope classes following [53]. As a result, the classes were: (1) flat < 10, (2) intermediate 10–20, and (3) steep > 20.

The human disturbance (which includes harvesting trees for fuel, wood, charcoal, timber, and house construction) was computed as the harvesting index. The harvesting index was measured by counting individual stumps, which reflected illegally logged trees, within the quadrat and calculated from the relative density of individual tree stumps. The relative density of stumps was computed as the sum of stump density divided by the total density (the sum of the logged stump and living individual trees). Stumps are a small portion of the trunk that remains after a tree with about 5 cm diameter is chopped down [54].

### Spatial data analysis

#### Land use/land cover data

The LULC types and Tag Image File Format (TIFF) data were obtained from a previously published article by Tadese, Soromessa [55]. They included area statistics for five different land cover types for the years 1987, 2002, and 2017 (Table 2).

**Table 2:** Area of LULC classes from 1987 to 2017 in MFBR adopted from Tadese et al. [46]

LULC classes	1987		2002		2017	
	Area (ha)	Area (%)	Area (ha)	Area (%)	Area (ha)	Area (%)
Forestland	196,761.6	84.4	188,413.7	80.8	181,504.9	77.8
Farmland	30,781.8	13.2	36,906.4	15.8	40,554.8	17.4
Grassland	3509.2	1.5	3079.6	1.3	3192.2	1.4
Settlement	2050.7	0.9	4744.3	2.0	7866.2	3.4
Water body	141.0	0.06	141.0	0.06	141.0	0.06
Total	233,254	100%	233,254	100%	23,3254	100%

**Fig. 3:**
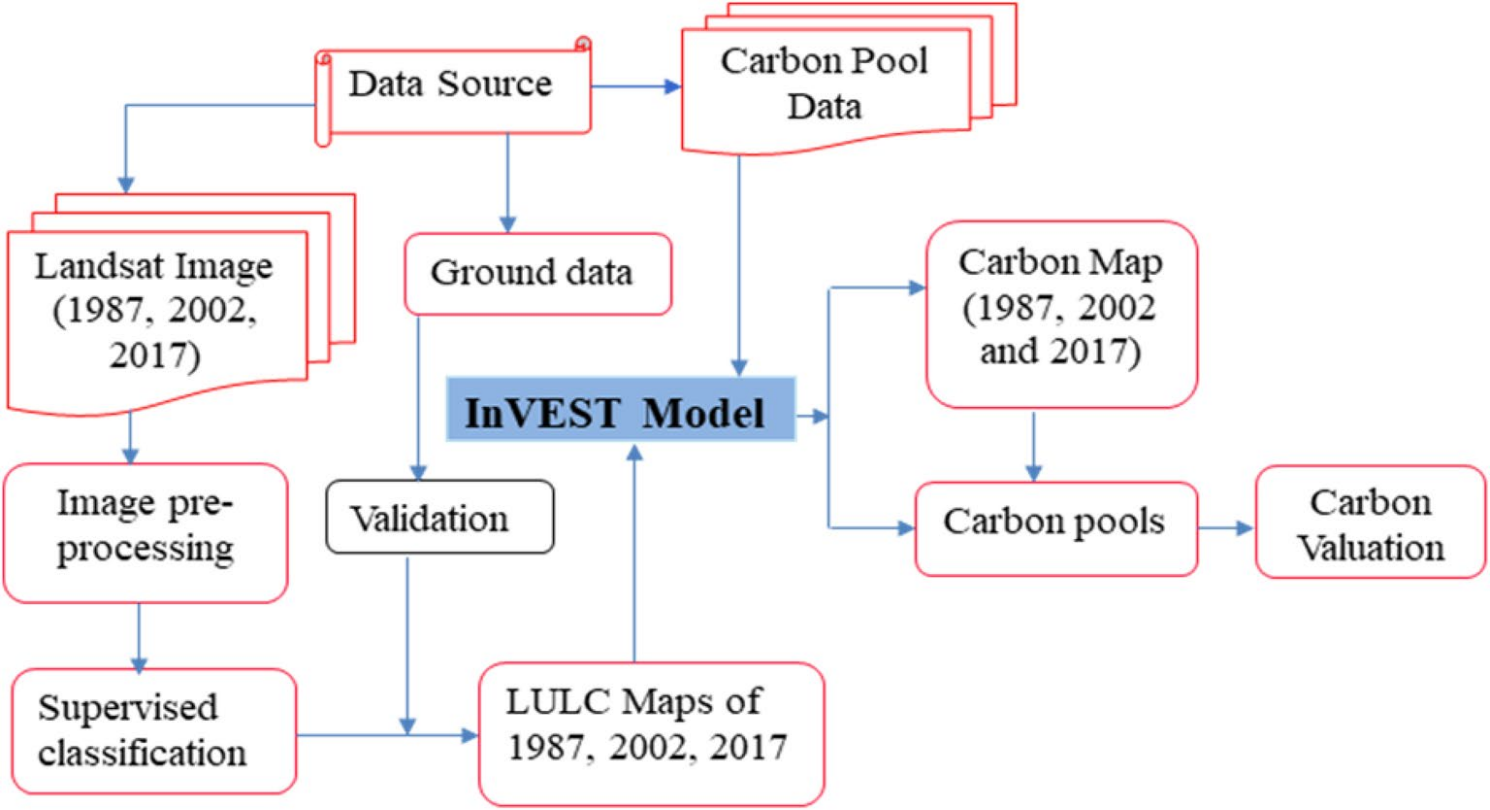
Flow chart of the methodology

The spatial distribution of carbon stock pools in different LULC types for each study year (forest land, farmland, and grassland) were analysed using the InVEST model (Fig. 3).

#### Carbon stock estimation using the InVEST model

The InVEST modelling framework is a set of open-source models for mapping and valuing the goods and ecosystems that produce the flow of services required to sustain life on Earth [21, 56, 57]. We customised the InVEST carbon stock mapping and sequestration model to assess the amount of total carbon stored in the five carbon pools (above-ground biomass, below-ground biomass, deadwood, litter, and soil organic matter) in different LULC classes of the study area. Carbon stock values were assigned to each LULC class for the selected years (i.e., 1987, 2002, and 2017) using field inventory data for forest land, farmland, and grassland.

To meet the model’s requirements, the LULC and carbon pool data sets were prepared and used as the primary input data to estimate carbon storage in each grid cell. Land use codes, the name of the LULC class, the amount of Above-Ground Biomass (AGB), Below-Ground Biomass (BGB), deadwood (DW), Litter, Grass, and Herbs (LGH), and Soil Organic Matter (SOC) are all included in the carbon stock data set in an MS Excel database. The LULC class is encoded with land-use codes in each row. Except for settlements and water bodies, which have zero carbon stock in all carbon pools, each column contains different attributes of the LULC type. Carbon in each pool was then combined across land-use types to estimate the total carbon storage.

#### Soil laboratory analysis

The soil samples were analysed in the Water Works Design and Supervision Enterprise laboratory (WWDSE) in Addis Ababa, Ethiopia. The Bouyoucos Hydrometer Method was used to determine the soil textures (expressed as a percentage of weight). It is a particle size analysis method that calculates the physical proportions of soil particles based on their settling rates in an aqueous solution [58]. Soil pH was determined using a pH meter and a 1:2.5 soil to water suspension potentiometric method [59]. The Micro-Kjeldahl [60] and Walkley and Black [61] methods were used to determine total nitrogen (N) and soil organic carbon, respectively. The Bray-I method was used to determine available phosphorus, and the absorbance of the Bray-I extract was measured in a spectrophotometer at an 882 nm wavelength [62]. Based on C and N concentrations, the Carbon to Nitrogen ratio (C/N) was calculated. The mass of each soil sample (MS) was determined using oven-drying set to 105 °C for 24 h to achieve a constant weight [49]. The volume of the Core Sampler (VC) was determined as VC = π r^2^h, where r is the radius and h is the height of the core sampler (VC = 3.14 × (2.5 cm)^2^ × 5 cm = 98.125 cm^3^).

#### Carbon stock and value analysis

Data analysis of various carbon pools measured in the forests was performed in R version 4.0.1. [63]. The AGB of trees was calculated using a previously published allometric equation in which the independent variables were trunk diameter (D, cm), height (H, m), and wood density (p, g cm^−3^) (predictors) [64].The Global Wood Density database was used to determine the wood density of different species [65]. The following formula [64] was employed to calculate the above-ground biomass with the BIOMASS package in R [66]:

1 $$\text{AGB} \,(\text{kg})={0.0673} * ({pD}^{2}{H})^{0.976},$$

where *AGB* is the above-ground biomass of trees (kg), *p* is the specific wood density (g cm^−3^), *D* is the trunk diameter at breast height (cm), and *H* is the total height of trees (m). The total AGB carbon for each quadrat was calculated as aggregate AGB carbon for all trees. Carbon stocks were determined for each quadrat and then extrapolated to tonnes per hectare. The carbon content in AGB is calculated by multiplying the default carbon fraction by 50% [67].

Below-ground biomass was estimated with the equation developed by [50]

2 $$\text{BGB}=\text{AGB} *0.2,$$

where *BGB* is below-ground biomass, *AGB* is above-ground biomass, 0.2 is the conversion factor (or 20% of AGB).

For standing deadwood (SDW) which has branches, the biomass was estimated using the allometric equation for the estimation of above-ground biomass [51].

For the remaining standing deadwood, the biomass was estimated using wood density and volume calculated from the truncated cone [51].

3 $$\text{Volume} \;{(\text{m})}^{3}=\frac{1}{3}{\uppi }{\text{h}\;{\text{r}_{1}^{2}}}+{{\text{r}}_{2}^{2}}+{\text{r}}_{1}*{\text{r}}_{2},$$

where *h* is the height in meters, *r*_1_ is the radius at the base of the tree, and *r*_2_ is the radius at the top of the tree.

4 $$\text{Biomass} = \text{Volume} \times \text{Wood} \; \text{density}\; (\text{from} \; \text{samples}).$$

The biomass of lying deadwood was estimated by the equation given below [51].

5 $$\text{LDW} =\sum_{i=1}^{n}\text{V}*\text{S},$$

where *LDW* is lying dead wood, *V* is volume, and *s* is the specific density of each density class.

The lying deadwood volume per unit area is estimated with:

6 $$\text{V}= {\pi }^{2} \left(\sum \frac{{\text{D}1}^{2}+{\text{D}2}^{2}}{8\text{L}}\right),$$

where *V* is the volume in m^3^/ha; *L* is the length of the line transect, and *D* is the diameter of the deadwood tree. The carbon content in AGB is calculated by multiplying the default carbon fraction by 50% [67].

The biomass in the pool of leaf litter, grass, and herbs was estimated using destructive sampling. Forest floor litter material (dead leaves, twigs, fruit, and flowers) was collected from a 1 m^2^ area. The living components, primarily grass and herbs, were harvested and weighed as well. Dry weight was determined in laboratory samples of the material. To estimate the biomass carbon stock of the litter, 100 g of fresh litter subsample was taken for laboratory use, and each sample was then dried in an oven at 105 °C for 24 h to obtain the dry weight [51].

The leaf litter, grass, and herbs (LGH) biomass per hectare was computed using the following formula:

7 $$\text{LHG} =\frac{\text{W}_{field}}{A} \times \frac{\text{W}_{subsample,\;dry}}{\text{W}_{subsample,\; wet}}\times \frac{1}{10,000},$$

where *LHG* is the leaf litter, herbs, and grass biomass (tonne ha^–1^), *W*_*field*_ is the weight of fresh leaf litter, herbs, and grass sampled destructively within area A (g), *A* is the size of the area where leaf litter, herbs, and grass were collected (ha), *W*_*subsample*_, _*dry*_ is the weight of oven-dried sub-sample of leaf litter, herbs, and grass taken to the laboratory for moisture content determination (g), *W*_*subsample*_, _*wet*_ is the weight of fresh sub-sample of leaf litter, herbs, and grass taken to the laboratory for moisture content determination (g).

Carbon stocks in litter biomass were calculated using the following formula:

8 $$\text{CL} = \text{LHG} * {\% \text{C}},$$

where *CL* is the total carbon stocks in litter in tonne ha^–1^, *LHG* is the leaf litter, herbs, and grass biomass (tonne ha^–1^) and *% C* is the carbon fraction determined in the laboratory [51].

The soil carbon stock was assessed in this study using the fine soil fraction to a depth of 30 cm. The following equation was used to calculate the bulk density (BD):

9 $${\text{BD}}=\frac{{\text{MS}}}{{\text{VC}}},$$

where *BD* is the bulk density (g cm^–3^), *MS* is the mass of the oven-dry soil (g) and *VC* is the volume of the core sampler (cm^3^).

The amount of carbon stored per hectare was calculated using the following formula, taking into account soil depth (cm), bulk density (g cm^–3^), and the percentage of soil organic carbon content (SOC), which is the recommended method [51].

10 $$\text{SOC} = \text{BD} \times \text{d} \times {\%\text{C}},$$

where *SOC stock* is the soil organic carbon stock per unit area (tonne ha^–1^), *BD* is the bulk density (g cm^–3^), *d* is the total depth of the sample (30 cm), and *% C* is the soil organic carbon concentration (ppm).

The carbon stock density of each stratum was calculated by aggregating the carbon stock densities of each stratum’s carbon pools using the formula in the following equation.

11 $$\text{C} \;(\text{LU}) = \text{C} \;(\text{AGB}) +\text{C} \;(\text{BB})+\text{C}\; (\text{DWB}) + \text{C}\;(\text{LHG})+\text{SOC},$$

where *C (LU)* is the carbon stock density for a land-use category (C t ha^–1^), *C (AGB)* is the carbon in above-ground tree components (C t ha^–1^), *C (BB)* is the carbon in below-ground components (C t ha^–1^), *C (DWB)* is the carbon in deadwood tree components (C t ha^–1^), *C (LHG)* is the carbon in the litter, herbs, and grass (C t ha^–1^), *SOC* is the soil organic carbon (C t ha^–1^).

Carbon was summed, and the total was then multiplied by 44/12 (3.67) to convert it into the carbon dioxide equivalent.

A chronological carbon storage change investigation was conducted at MFBR for the reference years 1987, 2002, and 2017 according to the method proposed by [68]. After calculating the carbon stock and value based on the previous, baseline year in the MFBR, change was analysed using the below equation.

12 $${\Delta} \text{C}=\frac{C_{Final\;year}-C_{initial\;year}}{C_{initial\;year}}*100\%,$$

where *∆C* is the percentage change in carbon, *C*_*final year*_ is the carbon stock in the final (recent) year, and *C*_*initial year*_ is the carbon stock in the initial years.

### Carbon market value estimation

#### Global voluntary market price

The global voluntary market price of carbon sequestration was compared using two data sources: the Global Voluntary Market Price (GVMP) and Tropical Ecosystems and Biodiversity (TEEB) database valuation. The carbon storage rate for the landscape is necessary to determine carbon sequestration (CO_2_e) in the GVMP set by different actors, such as the World Bank. The carbon storage rate (t ha^–1^) multiplied by 3.67 (44/12 = 3.67) is used to estimate CO_2_e [49]. Hence, the sequestered carbon (CO_2_e) is multiplied by the market price of carbon storage (4.40 USD/tCO_2_e) which was the carbon credit used in the Clean Development Mechanism (CDM) project under the Humbo forest rehabilitation in Ethiopia [69, 70]. To analyse the monetary value, the annual rate of change in the carbon price of 3% and the market discount rate of 7% was required to estimate carbon storage value. The total value of carbon stock has been estimated by the sum of each land-use type area multiplied by the monetary value of its carbon stock.

#### TEEB carbon valuation data

The Tropical Ecosystems and Biodiversity (TEEB) database (http://www.teebweb.org) contains the monetary value of carbon sequestration for various land-use types [71]. The TEEB data were collected from different parts of the biome and analysed using different methods such as direct market pricing, avoided cost, and benefit transfer [37, 38]. These valuation data were adapted to East Africa to compare the carbon sequestration values for MFBR (Table 3). The total carbon value was calculated by multiplying the area (ha) of each LUC type by its corresponding value of CO_2_e for that particular LULC type [39, 72].

**Table 3:** Carbon sequestration value for each LULC type in the TEEB database

No.	LULC	Carbon sequestration prices (USD/ha/yr)
1	Forest land	1229.79
2	Grazing land	297
3	Farmland	96

In other words, the value of Co_2_e obtained from TEEB multiplied by the LULC area yields the total market value of carbon. The carbon stock value data obtained from the TEEB database has been rearranged (sorted, summed, filtered by region, etc.) for supplementary analysis.

The carbon stock value was estimated based on two approaches. In the first approach, the carbon stock value was estimated using GVMP (4.40 USD in 2019), which is considered a discount rate (7%) and the annual rate of change in the carbon price (3%).

It was calculated using the following equation:

13 $$\text{TCV} = \text{CS}\; (5 \;\text{pools})\, \text{t}/\text{ha} * \text{Area}\; (\text{ha}) * \text{CP}\; ({\$}/\text{tonne}) - \text{DR}+\text{ARC},$$

where *TCV* is the total carbon value, *CS* is the carbon stock in five pools, *CP* is the carbon price per tonne, *DR* is the discount rate, and *ARC* is the annual rate of carbon price change.

In the second approach, the carbon stock value was estimated using the TEEB database, which contains carbon sequestration values for each LULC type (Table 3).

It was calculated using the equation below:

14 $$\text{TCV} = \text{CS}\; (5\; \text{pools})\, \text{t}/\text{ha} * \text{Area}\, (\text{ha}) * \text{CP}\; \text{of}\; \text{LUC} \;\text{type}\;({\$}/\text{tonne}),$$

where *TCV* is the total carbon value, *CS* is the carbon stock in five pools, *CP* is the carbon price for each LUC type per tonne.

### Statistical analysis

One-way ANOVA was used to determine whether there were significant differences between environmental and disturbance factors regarding carbon stocks in R software [63]. The statistical significance level was set at 5%. Pearson correlation analysis was used to examine the relationship between environmental-disturbance factors regarding carbon stocks. When the value of *r* approaches negative 1, the carbon stock and the independent variable (factors) are inversely proportional (carbon stock increases as the factors decrease). If *r* approaches positive 1, the carbon stock increases while the factors increase.

## Results

### Carbon stock in carbon pools

In the MFBR, the mean above-ground carbon (AGC) and below-ground carbon stocks (BGC) in the forest land were 272.57 and 54.97 t ha^–1^, respectively (Table 4). The minimum and maximum of the mean AGB carbon stock were 144.21 and 661, while BGB carbon stocks were 28.84 and 155.81 t ha^–1^ in MFBR, respectively. The distribution patterns of the BGB carbon stock showed similar trends to those of the AGC stock. The mean dead wood and litter, herbs, and grass carbon (LHGC) stocks were 3.04 t ha^–1^ and 0.05 t ha^–1^, respectively. The minimum and maximum deadwood carbon (DWC) stocks were 0.13 and 6.11 t ha^–1^, while litter, herbs, and grass LHGC stocks were 0.016 and 0.32 t ha^–1^, respectively. The mean soil organic carbon (SOC) stock was 176.26 t ha^–1,^ and the minimum and maximum SOC stocks were 116.96 and 280.31 t ha^–1^ respectively (Table 4).

**Table 4:** Total carbon stocks and CO_2_ sequestration (t/ha) in four study sites

Carbon pool	Study sites				
	Site I	Site II	Site III	Site IV	Mean TCS (MFBR)
AGC	269.7 ± 7.3	260.8 ± 8.5	277.9 ± 20.0	282.0 ± 15.1	272.57
BGC	53.9 ± 1.5	50.6 ± 1.6	55.6 ± 4.1	59.7 ± 4.1	54.97
DWC	2.4 ± 0.2	2.7 ± 0.1	3.3 ± 0.3	3.8 ± 0.27	3.04
LHGC	0.03 ± 0.01	0.04 ± 0.02	0.05 ± 0.01	0.08 ± 0.01	0.05
FoSOC	161.0 ± 2.1	166.2 ± 2.7	178.6 ± 5.1	199.3 ± 7.1	176.26
FaLSOC	128.6 ± 5.8	129.1 ± 4.1	131.1 ± 6.3	135.8 ± 2.7	131.16
GLSOC	145.7 ± 5.4	154.0 ± 6.7	148.0 ± 5.6	148.8 ± 4.1	149.09
TCS	761.3 ± 9.5	763.3 ± 10.5	794.5 ± 24.6	829.4 ± 26.2	787.14
CO_2_ Seq.	2794.0 ± 35.1	2801.5 ± 38.5	2915.7 ± 90.5	3043.9 ± 25.6	2888.79

Site I ≤ 1200, site II = 1200–1500, site III = 1500–1800, and site IV ≥ 1800 m a.s.l.

*AGC* above ground carbon, *BGC* below ground carbon, *DWC* dead wood carbon, *LHGC* litter, herbs and grass carbon, *FoSOC* forest soil organic carbon, *FaLSOC* farmland soil organic carbon, *GLSOC* grassland soil organic carbon, *TCS* total carbon stock

**Fig. 4:**
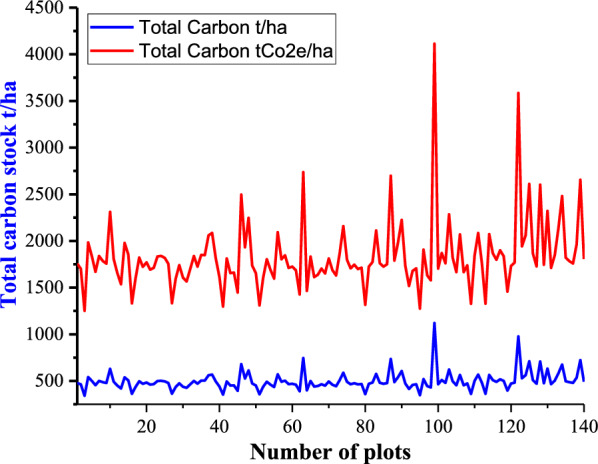
Total carbon stock in t ha^–1^ and tCO_2_e t ha^–1^ for each plot

The mean carbon stock in the carbon pool increased from site one to site four. The mean AGC stock varied among the four study sites of the MFBR, ranging from 260.8 ± 8.5 to 282.0 ± 15.1 t ha^–1^. The soil carbon pool has a significant contribution to the total carbon stock of MFBR. The SOC of MFBR fluctuated among the study sites; site four contributed the highest SOC (199.3 ± 7.1 t ha^–1^), followed by site one (178.6 ± 5.1 t ha^–1^). The smallest amount of soil carbon was obtained for site one (161.0 ± 2.1 t ha^–1^) (Table 4). The mean forest SOC stock in MFBR increased with elevation from study site one to four, ranging from 161.0 ± 2.1 to 199.3 ± 7.1 t ha^–1^, respectively, (Table 4). Similarly, the mean SOC for farmland and grassland increases with elevation and varies from 128.6 ± 5.8 to 135.8 ± 2.7 and from 145.7 ± 5.4 to 148.8 ± 4.1 t ha^–1^, respectively. In comparison, the total carbon stock stored in forest biomass was higher than in grassland and farmland in the MFBR. The overall mean total carbon stocks and sequestration for all LULC types were 787.14 t ha^–1^ and 2888.79 t CO_2_e ha^–1^, ranging from 761.3 ± 9.5 to 829.4 ± 26.2 t ha^–1^ for carbon stocks and from 2794.0 ± 35.1 to 3043.9 ± 25.6 t CO_2_e ha^–1^ for carbon sequestration, respectively, along the elevation gradient in MFBR (Fig. 4).

The total AGC stocks of five dominant species in four study sites are shown in (Table 5). In study site I, the total AGC stock of the first five species 119.4 t ha^–1^ (44.8%). The highest AGC stock was contributed by *Cordia Africana* (41.2 t ha^–1^) followed by *Combretum molle* (28.3 t ha^–1^) *and Lecaniodiscus fraxinifolius* (22.3 t ha^–1^). The first five species of the total AGC stock amounted to 138.9 t ha^–1^ (55.1%) in study site II. The highest AGC stock was found for *Fagaropsis angolensis* (45.9 t ha^–1^), followed by *Albizia grandibracteata* (30.1 t ha^–1^), and *Cordia africana* (25.4 t ha^–1^). The total AGC stock of the first five species was 120 t ha^–1^ (46%) in site III. The highest mean AGC stock was contributed by *Cordia Africana* (38.0 t ha^–1^), followed by *Ficus mucuso* (28.7 t ha^–1^) and *Croton sylvaticus* (21.5 t ha^–1^). The total AGC stock of the first five species contributed 90.3 t ha^–1^ (35.1%), in study site IV. The highest AGC stock was contributed by *Allophylus abyssinicus* (29.2 t ha^–1^), followed by *Prunus africana* (16.9 t ha^–1^), and *Ficus sur* (12.3 t ha^–1^) (Table 5).

**Table 5:** Mean above-ground carbon stocks in five dominant species in four study sites

Species	WD	DBH	H	Ind	AG-C	% AG-C
Site I						
*Cordia africana*	0.54	35.2	26.8	22	41.2	15.3
*Combretum molle*	0.73	29.1	30.8	14	28.3	10.7
*Lecaniodiscus fraxinifolius*	0.73	25.8	21.4	26	22.3	8.4
*Morus mesozygia*	0.72	23.7	19.3	8	15.4	5.8
*Manilkara butugi*	0.95	24.1	20.4	9	12.2	4.6
Site II						
*Fagaropsis angolensis*	0.57	44.8	37.2	12	45.9	18.2
*Albizia grandibracteata*	0.46	45.7	32.0	6	30.1	11.9
*Cordia africana*	0.54	33.2	24.5	43	25.4	10.1
*Mimus opslanceolata*	0.86	41.2	31.2	10	20.7	8.2
*Grewia mollis*	0.82	24.4	24.6	27	16.8	6.7
Site III						
*Cordia africana*	0.54	42.1	34.0	40	38.0	14.7
*Ficus mucuso*	0.44	28.2	21.9	14	28.7	11.1
*Croton sylvaticus*	0.64	26.2	27.8	20	21.5	8.3
*Apodytes dimidiata*	0.61	19.7	17.3	28	17.8	6.9
*Blighia unijugata*	0.56	23.6	21.8	42	13.0	5.0
Site IV						
*Allophylus abyssinicus*	0.61	28.3	25.0	34	29.2	11.3
*Croton macrostachyus*	0.52	26.8	25.8	8	21.1	8.2
*Prunus africana*	0.69	27.1	25.0	26	16.9	6.6
*Ficus sur*	0.41	32.1	25.3	10	12.3	4.8
*Trilepisium madagascariense*	0.50	28.7	24.4	49	10.8	4.2

*WD* wood density (g cm^–3^), *DBH* diameter at breast height (cm), *H* height (m), *Ind* individual number, *AG-C* above-ground carbon (t ha^–1^)

**Fig. 5:**
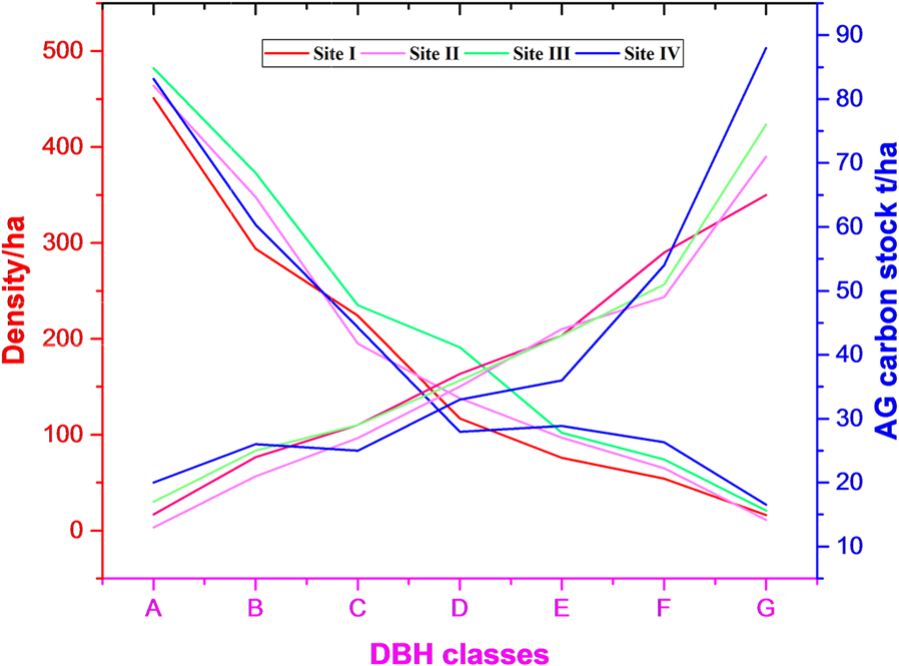
Density and AG carbon stock along DBH classes in MFBR. A ≤ 10, B = 10.1–20, C = 20.1–30, D = 30.1–40, E = 40.1–50, F = 50.1–60, and G ≥ 60 cm

In this study, DBH classes are directly related to the AGC stock while inversely related to trunk density per hectare. The trunk density of smaller-sized classes is higher than that of larger-sized classes, although they contribute a smaller amount of AGC stock per hectare. Moreover, the larger trunk diameter classes (DBH ≥ 40) showed higher AGC stock in site I (60.9%), site II (63.1%), site III (61.4%), and site IV (63.1%) as compared to smaller trunk diameter classes (DBH ≤ 40) (Fig. 5). Therefore, the amount of AGC stock increased with DBH, which indicated that harvesting larger-sized trees leads to carbon stock reduction. The density per hectare decreases with an increase in DBH classes.

Above-ground carbon stock showed a strong positive correlation with DBH classes(*r* = 0.85 and *P* = 0.05), while density per hectare showed a strong negative correlation with DBH classes (*r* = − 0.89 and *P* = 0.05).

### Carbon stock in land use/land cover

Above-ground biomass carbon (272.57 t ha^–1^) had the highest carbon pool in the forest land followed by SOC (176.26 t ha^–1^), while LHG biomass (0.05 t ha^–1^) had the lowest carbon pool (Table 6). The shares of carbon pools in forest land were the following: the AGC (53.77%), BGC (10.84%), DWC (0.59%), LHGC (0.009%), and SOC (34.77%). In general, the highest contribution came from AGC, followed by SOC, BGC, and DWC. In comparison, SOC stock in forest land (176.26 t ha^–1^) was higher than for grassland (149.09 t ha^–1^) and farmland (131.16 t ha^–1^) (Table 6). Thus, the contributions of SOC were (22.39%), (18.94%), and (16.66%) in forest land, grazing land, and farmland, respectively. The AGC, BGC, and DWC pools were not estimated on grassland and farmland due to the absence of trees exceeding 5 cm DBH in the study plots (Table 6).

**Table 6:** Carbon pools by land use/land cover (t/ha)

LULC	AGC	BGC	DWC	LHGC	SOC	Total
FoL	272.57	54.97	3.04	0.05	176.26	506.88
FaL	0	0	0	0	131.16	131.16
GL	0	0	0	0.001	149.09	149.09
Set	0	0	0	0	0	0
WB	0	0	0	0	0	0
Ave				0.055	152.17	152.225

*AGC* above ground carbon, BGC below ground carbon, *DWC* dead wood carbon biomass, *LHGC* litter, herbs, and grass carbon, *SOC* soil organic carbon, *FoL* forestland, *FaL* farmland, *GL* grassland, *Set* settlement, *WB* water body, *Ave* average

Above-ground carbon, BGC and DWC amounted to about 34.62%, 6.98%, and 0.38% of forest land storage, respectively. The share of LHGC was 0.006% in the forest land and grassland. Likewise, there was no significant contribution from the water body and settlement (Table 6).

### Effects of land cover change on carbon stock

**Fig. 6:**
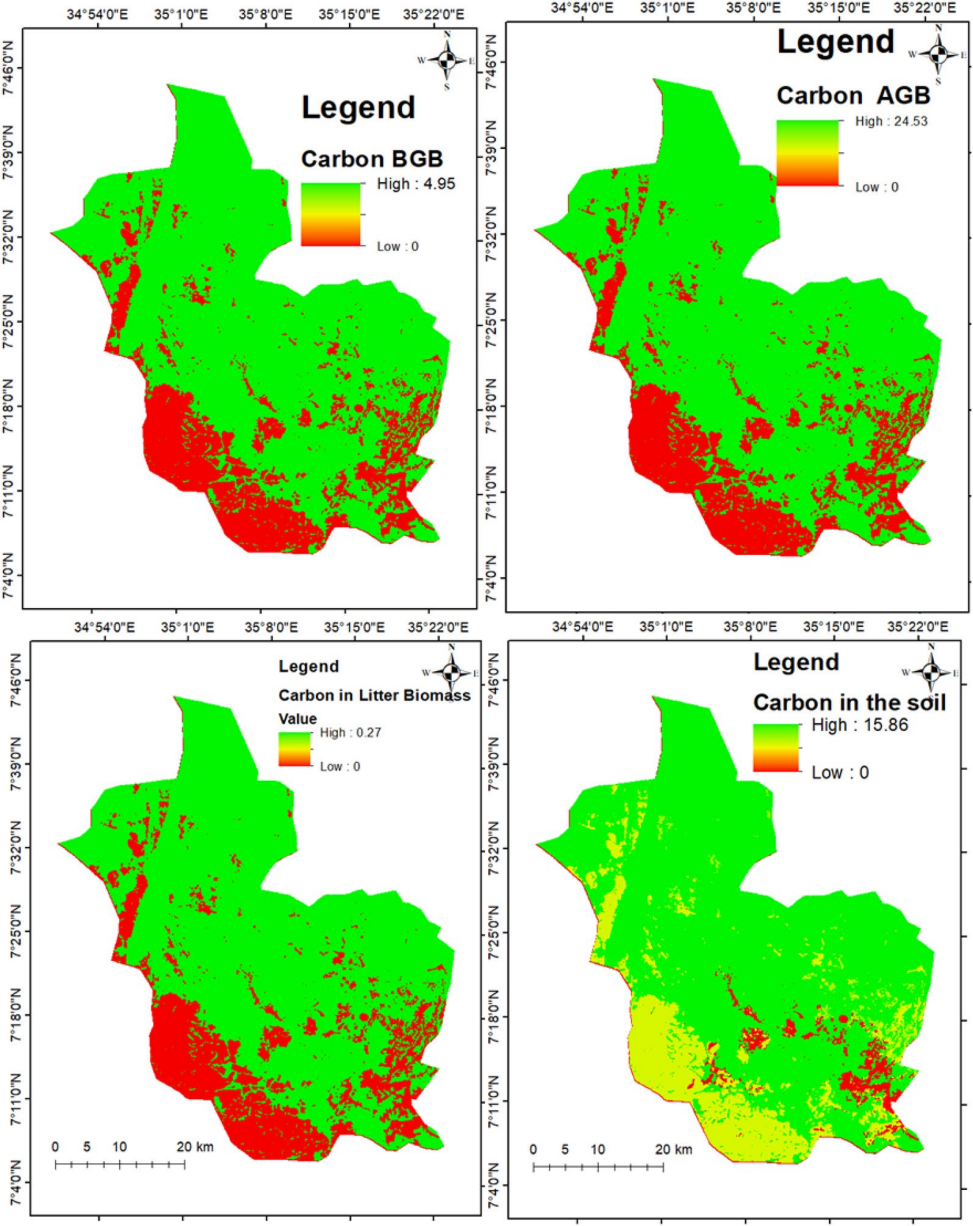
Spatial and temporal description of carbon stocks for the reference years

**Fig. 7:**
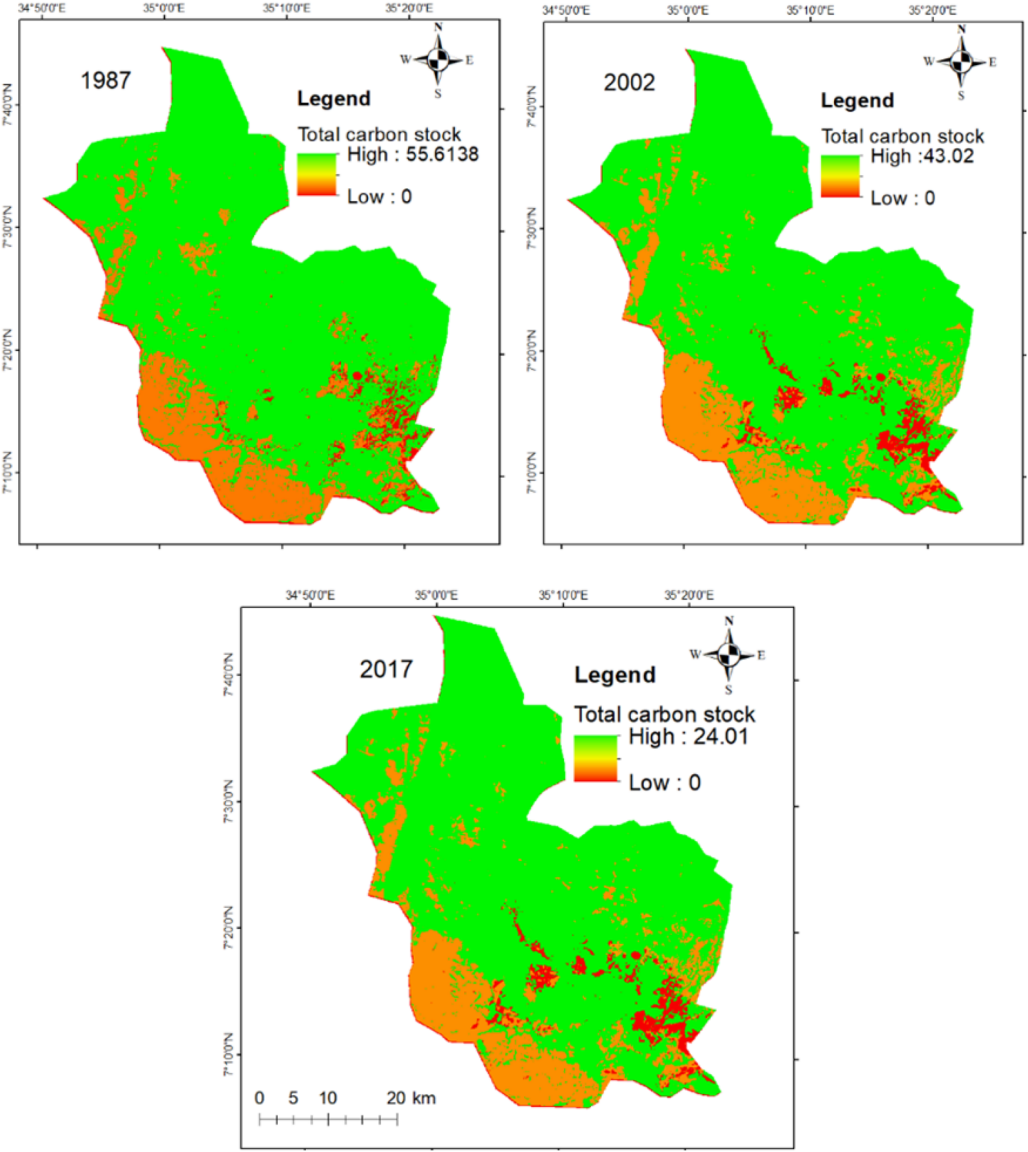
Spatial distribution of carbon pools per pixel

The LULC affected the carbon stock during the 1987 to 2017 period in MFBR (Table 7). The maximum carbon stock was found in forest land (99.73 million tonne) followed by farmland (4.03 million tonne), whereas the lowest was identified in grassland (0.52 million tonne) in 1987 of MFBR (Table 7). Similarly, the maximum carbon stock was shown in forest land (92.01 million tonne) followed by farmland (5.32 million tonne) whereas the lowest was identified in grassland (0.47 million tonne) in 2017 of MFBR. Based on the InVEST carbon model results, the conversion of forest land and grassland into farmland led to a reduction of carbon stock in MFBR (Figs. 6 and 7). The chronological investigation indicated that the carbon stock declined by 7.73 million tonne in forest land from 1989 to 2017, while the average carbon stock was reduced by 2.16 million tonne with an annual loss of 0.07 million tonne. The drop in the carbon stock is due to the reduction of forest land and grassland from 1987 to 2017 in MFBR.

**Table 7:** Carbon storage and its changes in the reference years (million t/ha and CStCO_2_e/ha)

LUC	1987		2002		2017		Change (1987–2017)	
	CS t ha^–1^	CStCO_2_e ha^–1^	CS t ha^–1^	CStCO_2_e ha^–1^	CS t ha^–1^	CStCO_2_e ha^–1^	CS t ha^–1^	CStCO_2_e ha^–1^
FoL	99.73	366.02	95.50	350.49	92.01	337.64	− 7.73	− 28.28
FaL	4.03	14.82	4.84	17.76	5.32	19.52	1.28	4.70
GL	0.52	1.92	0.45	1.68	0.47	1.75	− 0.04	− 0.17
Ave	34.76	127.58	33.60	123.31	32.59	119.63	− 2.16	− 7.9

CS t ha^–1^: carbon stock ton per ha; CStCO_2_e ha^–1^: carbon stock ton carbon dioxide equivalent per ha; FoL: forestland; FaL: farmland; GL: grassland; Ave: average

In forest land, the total carbon stock shrunk from 366.02 million tonne CO_2_e in 1987 to 337.64 million tonne CO_2_e in 2017 of MFBR. Forest land and grassland cover declined with 6.6% and 0.1%, respectively, which led to a reduction of 28.38 million and 0.17 million tonne CO_2_e in the previous 30 years respectively of MFBR (Table 7). The average carbon stock was diminished by 7.9 million tonne CO_2_ with an annual loss of 0.26 million tonne CO_2_, which is due to the reduction of forest land and grassland from 1987 to 2017 in MFBR (Table 7). In general, changing LULC classes reduce vegetation cover, which directly contributes to increased or reduced carbon sequestration and carbon market value.

### Carbon storage valuation

The global voluntary market price analysis showed that the average carbon sequestration was reduced from $5.55 billion in 1987 to $5.21 billion in 2017 in MFBR. In other words, the mean carbon value shrunk $0.011 billion t/ha/year over the previous 30 years. Forest land was the most important carbon-sequestering land-use class. However, the value of carbon sequestration decreased by $0.071 billion t/ha/year from $16.84 billion in 1987 to $14.70 billion in 2017 (Table 8).

**Table 8:** The estimated carbon storage valuation using GVMP and TEEB in each LULC in MFBR (billion USD)

LULC	1987		2002		2017		Change (1987–2017)	
	TEEB	GVMP	TEEB	GVMP	TEEB	GVMP	TEEB	GVMP
FoL	450.1	16.84	431	15.26	415.2	14.7	− 34.9	− 2.14
FaL	4.4	0.06	5.36	0.07	5.87	0.08	1.47	0.02
GL	0.18	0.0087	0.16	0.007	0.17	0.008	− 0.01	− 0.0007
Ave	151.56	5.55	145.48	5.37	140.39	5.21	− 11.17	− 0.34

*GVMP* global voluntary market price, *TEEB* Tropical Economics of Ecosystems and Biodiversity, *FoL *forestland, *FaL* farmland, *GL* grassland, *Ave* average

According to the carbon sequestration monetary value analysis of TEEB, the mean value of carbon sequestration went down from $1515.62 billion in 1987 to $1403.89 billion in 2017. The TEEB carbon sequestration value estimation ($1403.89 billion) is greater than that of GVMP ($5.21 billion) in 2017 (Table 8). This significant carbon value variation between GVMP and TEEB indicated a gap in carbon value estimation methods. Furthermore, the use of different methods of carbon pricing led to uncertainty in the estimation of carbon sequestration value.

Based on the estimation of TEEB and GVMP, carbon sequestrations for forest and grassland values have been drastically reduced as a result of human disturbances like vegetation. The TEEB and GVMP analyses estimated the carbon value of forest land to decline by $34.9 billion (7.75%) and $2.14 billion (12.70%), respectively, while grassland declined by $0.01 billion (5.55%) and $0.0007 billion (8.04%), respectively. Moreover, the average TEEB and GVMP valuation of carbon sequestration in MFBR declined by 11.17% and 0.34%, respectively (Table 8).

### Effects of environmental and disturbance factors on carbon stocks in forests

Based on the one-way ANOVA analysis, the harvesting index, elevation, slope, soil pH, total nitrogen, and phosphorus had a significant influence on carbon sequestration stock (*P* < 0.05) (Table 9).

**Table 9:** One-way ANOVA analysis of impact factors associated with carbon storage

Impact factors	df	Mean sq	F value	P-value
H-index	1	17018.2	3350	4.25E+06***
Elevation	1	887619.7	8391	5.99E+12***
Slope	1	16976.7	3371	2.38E+09***
Soil pH	1	17020.6	3348	3.79E+05***
TN	1	15424.1	3434	6.90E+07***
P	1	14216.2	3257	2.37E+04***

*df* degree of freedom

***P-value: – 0.001 indicates significant impact on carbon storage

Pearson correlation (r) tests exhibited both positive and negative relationships between environmental and disturbance factors with carbon stock in MFBR (Table 10). The AGC stock showed a positive relationship with SOC (r = 0.10), elevation (r = 0.08), TN (r = 0.31), and P (r = 0.11), while a significant negative relationship with the harvesting index (r = − 0.21) and pH (r = − 0.09).

**Table 10:** Pearson’s correlation coefficient matrix for environmental and disturbance factors (N = 8) in MFBR

Variables	AGC	SOC	HI	Ele	Slo	pH	TN	P
AGC								
SOC	0.10^ns^							
HI	− 0.21*	− 0.13^ns^						
Ele	0.08^ns^	0.39***	0.06^ns^					
Slo	− 0.04^ns^	− 0.03*	− 0.09^ns^	0.27***				
pH	− 0.09**	− 0.22**	− 0.02^ns^	− 0.69***	− 0.09**			
TN	0.31***	0.27***	− 0.31**	0.10^ns^	− 0.12^ns^	− 0.21***		
P	0.11^ns^	0.14^ns^	− 0.05^ns^	0.06^ns^	− 0.31*	− 0.16**	0.19***	

The magnitude indicates the degree of correlation and positive signs indicate positive correlation and negative signs indicate inverse relation

*ns* no significance, *N* number of variables, *MFBR* Majang Forest Biosphere Reserves, *AGC* above ground carbon, *SOC* soil organic carbon, *HI* harvesting index, *Ele* elevation, *Slo* slope, *TN* total nitrogen, *P* available phosphorus

*p < 0.05, **p < 0.01, ***p < 0.001

Above-ground carbon stock showed a weak positive correlation with elevation, while based on linear mixed-effect model regression showed an increase in elevation with a decrease of AGC in MFBR. SOC showed a significant positive correlation with elevation (r = 0.39) and TN (r = 0.27), but a significant negative relationship with slope (r = − 0.03), and pH (r = − 0.22). Similarly, the harvesting index showed a negative relationship with slope (r = − 0.09), pH (r = − 0.02), TN (r =− 0.31), and P (r = − 0.05) and a positive correlation with elevation (r = 0.06). Soil pH showed a significant negative relationship with TN (r = − 0.21), and P (r = − 0.16) (Table 10).

The linear mixed regression model analysis revealed that the elevation was not significant as random effect and the variance of random effect contribution in total corbon t/ha were 3.27%. In the fixed effects analysis, only nitrogen was significant fixed effect on total carbon (conditional R^2^ = 0.073, mariginal R^2^ = 0.042, P < 0.05) while the other randon effects were no significant effect on total carbon t/ha. Based on the model output, nitrogen increase the total carbon t/ha by 96.79 with Intercept (389.57). The final model of nitrogen with responses is TCS = 389.5786 + 96.79 TN (Table 11).

**Table 11:** Chi-square tests on fixed effects as response to total carbon tone per hectare

Fixed effects	Chisq	Df	Pr (> Chisq)
H. index	0.163	1	0.686
Slope	0.925	1	0.336
Soil. pH	0.928	1	0.335
TN	7.196	1	0.007**
P	0.493	1	0.482

*HI* harvesting index, *Slo* slope, *TN* total nitrogen, *P* available phosphorus

**p < 0.01

## Discussion

### Carbon stock in carbon pools and land use/land cover

The results of this study on carbon stocks show the importance of biosphere reserves for climate change mitigation. The study has confirmed a diverse variation in LULC and carbon stock pools along the elevation gradient in MFBR. For instance, the mean carbon stock in the LULC carbon pool increases along the elevation gradient (from site I to site IV) (Table 4). This finding is similar to the earlier finding that reported a positive relationship between elevation and carbon stock [73].

In comparison, the total carbon stock stored in forest land was higher than on grassland and farmland in MFBR (Table 4). Higher carbon stocks in forest land may be due to more vegetation cover and plant material decomposition [74]. Chuai, Huang [75] demonstrated that forest land also releases and absorbs huge amounts of carbon into and out of the atmosphere. Moreover, LULC conversion is the most important factor that causes the reduction and transformation of carbon sequestration in terrestrial ecosystems [76].

The AGC stock varied among study sites in MFBR (Table 4). The highest AGC stock was identified in study site IV (282 billion t ha^–1^), while the lowest (269.7 t ha^–1^) was in study site I of MFBR (Table 4). These results are consistent with carbon sequestration in the tropical Afromontane forest of Ethiopia (107–285) [53, 77–80] and in other tropical forests (170–271) [81–83]. This carbon stock difference may be related to DBH, height, and basal area of a tree. The variations in carbon sequestration at local, regional, and national levels could be the effect of human disturbances and environmental factors in the study site of MFBR.

Similarly, the mean SOC stock (0–30 cm soil depth) was 176.26 t ha^–1^ in MFBR. The highest SOC stock was found in study site IV (199.3 t ha^–1^), while the lowest was in study site I (161 t ha^–1^) of MFBR (Table 4). The mean SOC in MFBR was higher than earlier estimated carbon stock in other tropical forests (121–123 t ha^–1^) [84], a forest in Colombia (96 t ha^–1^) [85], Singapore (110 t ha^–1^) [86], the Humbo forest of Ethiopia (168 t ha^–1^) [87], and the Awi Zone of Ethiopia (149.2 t ha^–1^) [53]. Nevertheless, the mean SOC stock of MFBR was found to be lower than the SOC stock of tropical Afro-montane forests (194 to 288 t ha^–1^) [88].

The total carbon stocks varied from 487.04 to 544.87 t ha^–1^ in the study sites (Table 4), which is almost similar to the results quantified in the Adaba-Dodola community forest (507 t ha^–1^) [89], Gerba-Dima moist Afromontane forest (508.9 t ha^–1^) [78] in Ethiopia, and IPCC (130–510 t ha^–1^) [90]. Similarly, the mean total carbon stock of MFBR is higher than other findings in the Sheka Forest (461 t ha^–1^) [91], Humbo Forest (213.43 t ha^–1^) [87], and Singapore (337 t ha^–1^) [86]. This difference could be due to the existence of diverse tree species, elevation, human disturbance, climate, and microbial activities. Moreover, the comparison of five carbon stock pools with other tropical forests studies were showed in Table 12.

**Table 12:** Comparison of carbon stock with other tropical forests studies

Study area	Carbon stock in different pools (t ha^−1^)						Source
	AGBC	BGBC	DWC	LHGC	SOC	TCS	
Anshirava forest	180.18	77.51	1.36	2.69	111.43	338.18	Fikirte et al. [92]
Awi forests	191.7	38.24	–	–	149.3	380.8	Gebeyehu et al. [53]
Bangladesh forest	96.5	14.6	–	4.2	168.1	283.4	Ullah and Al-Amin [93]
Central Africa	168.6	39.5	–	–	–	208.1	Ekoungoulou et al. [94]
Egdu forest	278.08	55.62	–	3.47	277.56	614.73	Adugna et al. [95]
Gedo forest	281	56.1	2.37	0.41	183.7	523.6	Hamere et al. [80]
Gerba Dima forest	243.8	45.9	4.64	0.03	292.1	586.7	Abyot et al. [78]
Gesha-Sayilem forest	164.5	32.9		1.27	137.67	362.4	Admassu et al. [96]
Majang Forest	272.57	54.97	3.04	0.05	176.26	506.88	Present study
Sheka Forest	176.3	44	14.7	–	233	461	Ayehu et al. [91]
Singamba forests	142.3	38.45	–	–	–	175.82	Mattia and Sesay [97]
Tara Gedam forest	306.4	61.5	–	0.9	274.3	643.1	Mohammed et al. [98]
Tulu Lafto	218.4	43.5	6.2	2.4	128.9	399.4	Fekadu et al. [99]
Upper Omo-Gibe	185	37	–	32	178	432	Abreham et al. [77]
Usambra Forest	427	85.4	418	–	–	930.4	Munishi and Shear [100]
Wujig-Waren forest	65.8	11.4	–	2.25	102.3	181.78	Negasi et al. [101]

*AGBC* carbon storage in above-ground biomass, *BGBC* carbon storage in below-ground biomass, *DWC* carbon storage in dead wood biomass, *LHGC* carbon storage in litter, herbs, and grass biomass, *SOC* soil organic carbon

In the study sites, dominant species with higher basal areas demonstrated the highest carbon stock (Table 5) Individual plant species with higher DBH values contribute significantly to carbon sequestration in MFBR, while their extinction has a significant impact on biomass, carbon sequestration, and carbon trading. Deforestation and forest degradation also have an impact on the amount of carbon sequestered in larger trees with larger diameters [102]. The AGC and BGC in the study sites of MFBR were higher than the carbon value that was quantified by IPCC [67, 90]. This difference in AGC might be associated with the greater tree height, DBH, and basal area in MFBR.

#### Effects of land use/land cover change on carbon stock

This study shows how the carbon stock pool (AGC, BGC, DWC, LHGC, and SOC) is affected by LULC change in the study period (1987–2017) (Table 6; Fig. 7). Forest land showed higher carbon stock as compared to grassland and farmland in MFBR (Table 7). Similarly, other findings indicated higher carbon stock in forest land as compared to other LULC categories [101, 103]. This significant difference in carbon stock across land cover categories could be due to the difference in tree size and trunk density per hectare. Furthermore, lower carbon stock was found in farmland that has been altered by intensive subsistence cultivation, deforestation, or anthropogenic disturbance that affected the tree, shrub, and herb growth [104].

**Fig. 8:**
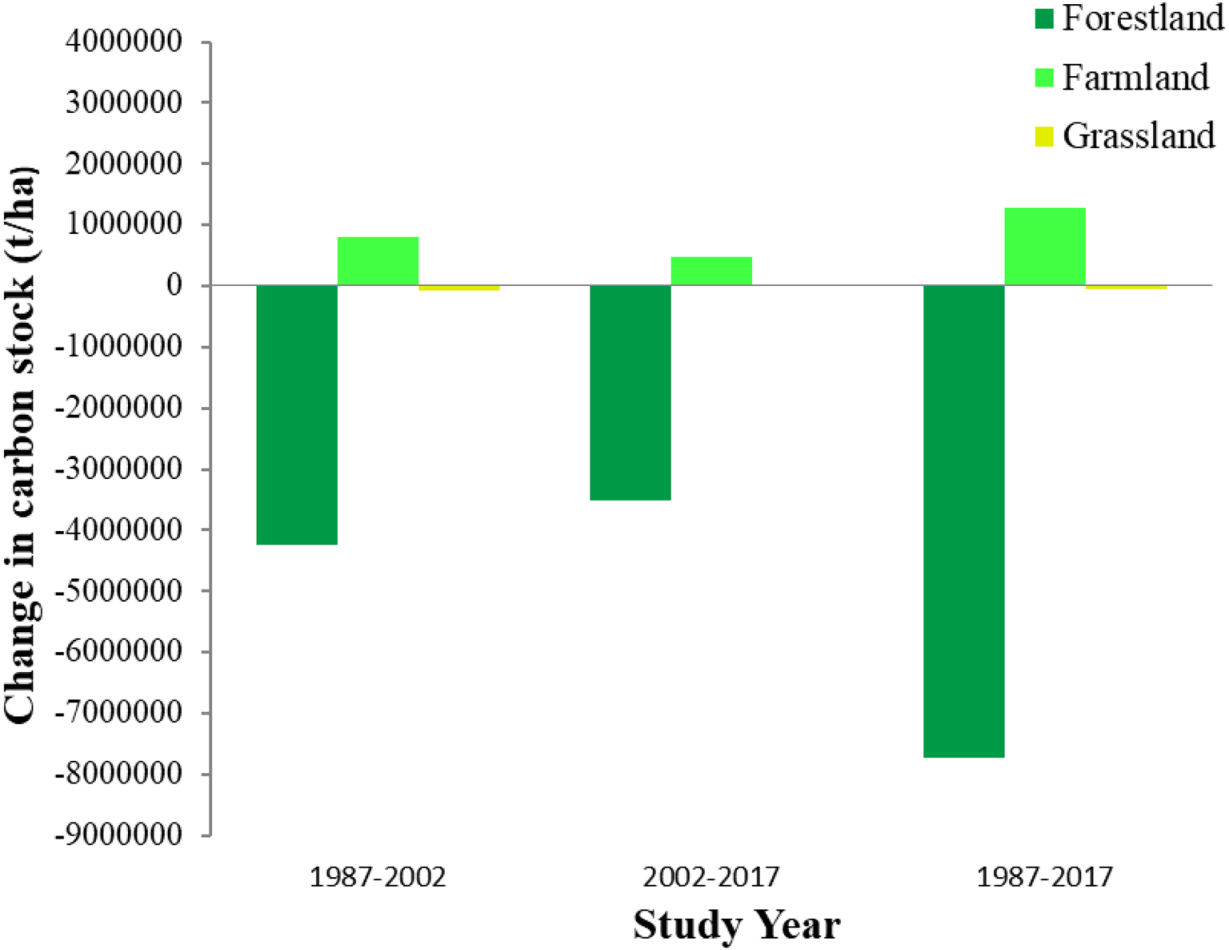
Carbon storage in MFBR over the last 30 years (1987–2017)

The InVEST carbon model results showed that the conversion of forest land and grassland into farmland leads to a reduction in carbon stock in the study area (Fig. 8). The chronological investigation indicated that the highest carbon stock was reduced in forest land among all LULC types. Carbon stock reduction was identified in all carbon pools as a result of forest land and grassland being converted into farmland in the study period (Fig. 7). This reduction is similar to phenomena in other reports [105–107]. This could be due to the expansion of settlements (urban and rural), agriculture expansion, and population pressure, which lead to deforestation and forest degradation [108, 109]. The increased urbanisation or settlement enlargement occurs at the expense of other LULC categories like farmland, forest land, and grassland [110, 111].

### Carbon storage valuation

Effective carbon stock valuation is highly relevant to the successful management of climate change impacts. It is also important for evaluating the relative advantages of climate adaptation activities and mitigation measures over time. The global voluntary market price analysis showed that the mean carbon sequestration value declined from 1987 to 2017 (Table 8). This carbon stock value reduction is linked to the change from forest cover to other LULC types, which is consistent with other findings [77].

Similarly, according to the carbon sequestration monetary value analysis of TEEB, the mean value of carbon sequestration dropped from 1987 to 2017. The TEEB carbon sequestration value estimation is greater than that of GVMP in all study periods for MFBR (Table 8). This significant carbon value variation between GVMP and TEEB indicated a gap in carbon value estimation methods. Furthermore, using different methods of carbon pricing led to uncertainty regarding the carbon sequestration value [112]. In addition, according to the estimation of TEEB and GVMP, forest and grassland carbon sequestration values have drastically shrunk as a result of such human disturbances as deforestation. Moreover, the average TEEB and GVMP valuation of carbon sequestration in MFBR declined (Table 8).

### Effects of environmental and disturbance factors on carbon stocks in forests

The relationship between carbon and environmental and disturbance factors has become more and more important in understanding the carbon sequestration cycle. In this study, environmental and anthropogenic factors highly influence forest cover and carbon sequestration in the pools. Accordingly, the variation in carbon stock was closely related to environmental and human disturbance. Elevation, slope, and harvesting index are important environmental and disturbance factors resulting in major differences in carbon stock among study sites in MFBR (Table 10).

The harvesting index and slope were also among the environmental factors that affected the variability of carbon in the pools. Carbon stock pools increase with decreasing slope, which may be related to the moisture and soil properties of the study sites. Furthermore, tree harvesting was the primary factor responsible for the decrease in biomass and carbon stocks. This shows that clear-cutting contributes to higher carbon emissions into the atmosphere [113]. As a result, forest conservation and sustainable management help reduce carbon emissions and keep biomass and carbon in carbon pools [114, 115].

The correlation between elevation and AGC and SOC stocks were negative and positive respectively. This finding is similar to an earlier study that stated a positive relationship between elevation and SOC [116]. The positive correlation between SOC and elevation may be due to a lower temperature and increasing moisture content with increasing elevation [88, 117]. The rate of organic matter decomposition is sluggish in low temperatures, which leads to reduced microbial activities, thus assisting the increments of soil organic matter and thicker litter layer development [118]. High organic matter content in soils at higher elevations has also been reported in other Afromontane forests of Ethiopia [119, 120]. This situation leads to a reduction in CO_2_ release from the soil, which in turn increases soil organic carbon stocks.

Slope with AGC and SOC stocks had a negative correlation. Greater slope with decreasing soil moisture resulted in decreased vegetation cover, hence a decline in AGC and SOC stocks. Similarly, the correlation between the harvesting index with AGC and SOC stocks was negative. This indicated that the harvesting index (selective exploitation) has a significant impact on AGC and SOC stocks. Thus, illegal harvesting focused on big trees for timber production leads to a reduction of carbon stocks, which is consistent with other findings [121, 122].

## Conclusions

In this study, the results showed high carbon stocks in MFBR, which is higher than other findings in moist Afromontane forests in Ethiopia. As regards carbon pools, the mean AGC and SOC stocks were shown to be higher than other pools in MFBR. The total carbon stock and economic value for the 2017 LULC data are lower than for the 1987 LULC data. The conversion of forest land and grassland into farmland reduces the carbon stock and its economic value in MFBR.

Forest cover and carbon sequestration in the pools are highly influenced by environmental and anthropogenic factors. Above-ground carbon stock showed a strong positive correlation with DBH classes (*r* = 0.85 and *P* = 0.05), while density per hectare showed a strong negative correlation with DBH classes (*r* = − 0.89 and *P* = 0.05). Accordingly, the variation in carbon stock was closely related to environmental and human disturbance. Elevation, slope, and harvesting index are important environmental and disturbance factors resulting in major differences in carbon stock among the study sites in MFBR. Therefore, the gradual reduction of carbon stocks in connection with LULC change calls for urgent attention to implement successful conservation and sustainable use of forest resources in biosphere reserves.

## Data Availability

The available data can be upon request the Corresponding Author.
